# Propensity for resistance development in the invasive berry pest, spotted‐wing drosophila (*Drosophila suzukii*), under laboratory selection

**DOI:** 10.1002/ps.7139

**Published:** 2022-08-31

**Authors:** Carrie Deans, William D Hutchison

**Affiliations:** ^1^ Department of Entomology University of Minnesota St Paul MN USA

**Keywords:** insecticide, spinosyn, pyrethroid, vinegar fly, LC_50_, dose–response

## Abstract

**BACKGROUND:**

Over the past 14 years, the invasive vinegar fly, spotted‐wing drosophila (*Drosophila suzukii*), has become one of the most damaging fruit pests in the United States. With regional economic losses estimated as high as $500 million for moderate infestations, *D. suzukii* control represents an often‐untenable cost to growers. Management relies heavily on chemical control, which may be applied up to nine times in one season. The widespread use of chemical controls has led to concerns about insecticide resistance, and resistant field populations have already been documented in California and Michigan.

**RESULTS:**

We cultured sub‐populations of three different Minnesota field populations of *D. suzukii* in the laboratory and exposed them to increasing concentrations of two commonly‐used insecticides, zeta‐cypermethrin (pyrethroid) and spinetoram (spinosyn). Over the exposure period, the sub‐populations experienced an 8‐ to 45‐fold increase in insecticide concentration. We saw significant increases in the median lethal concentration (LC_50_) values of one sub‐population exposed to zeta‐cypermethrin and one exposed to spinetoram. Across the spinetoram exposures, we also observed significant reductions in the top mortality values for three different sub‐populations.

**CONCLUSION:**

Our results suggest that field populations of *D. suzukii* can develop resistance to zeta‐cypermethrin and spinetoram in short periods of time under laboratory selection but that resistance to spinosyns occurs more readily than to pyrethroids. These results support other studies that have documented spinosyn resistance in field populations and in laboratory selections. Resistance evolution to spinosyns is a particularly important issue, as they represent one of few organic insecticide options for *D. suzukii*. © 2022 The Authors. *Pest Management Science* published by John Wiley & Sons Ltd on behalf of Society of Chemical Industry.

## Graphical abstract

We found evidence for resistance to zeta‐cypermethrin in one sub‐population and to spinetoram in three sub‐populations, indicating that resistance can develop quickly and is more likely for spinetoram than zeta‐cypermethrin.

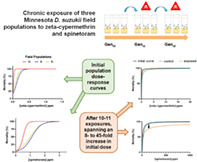

## 1 INTRODUCTION

Spotted‐wing drosophila (*Drosophila suzukii*), a vinegar fly native to southeast Asia, has become a recent invasive pest throughout Europe, North America, South America, and Africa.^1^, ^2^, ^3^, ^4^ Since its arrival in the continental United States in California in 2008, it quickly spread to the East coast by 2011 and became further established throughout the Midwest by 2013.^3^, ^5^, ^6^ *Drosophila suzukii* is a highly polyphagous species that feeds on several economically important berry and stone fruit hosts, but it can also utilize several native plant hosts. It occupies a unique niche by laying eggs in unripe intact fruits, a capability made possible by females possessing a serrated ovipositor. In this way, *D. suzukii* is able to utilize fruits that are unavailable to other vinegar fly species. *Drosophila suzukii* has become established in fruit‐producing regions in invaded areas, incurring significant costs to growers, both in yield reductions and in added costs of control. Though infestation rates vary, yield losses can be as high as 80–100% in some areas.^7^ Even at infestation rates of 20%, reports of potential economic losses of $500 million have been estimated for California, Oregon, and Washington alone.^7^ Additionally, the costs of chemical control ranges from $9 to $88 per acre, with 4–9 applications being required throughout the season depending on the crop.^8^, ^9^, ^10^

Chemical control is the primary method for *D. suzukii* management, which relies largely on broad‐spectrum contact insecticides, including pyrethroids, organophosphates, spinosyns, carbamates, and pyrazoles, as well as pyrethrin and spinosads as organic options.^11^, ^12^ Overall, chemical controls are effective,^13^ but reduced susceptibility has been documented in some field populations. In 2019, Gress and Zalom^14^ recorded spinosad resistance ratios of 4.3 to 7.7 for a *D. suzukii* population collected from a commercial raspberry operation in Watsonville, California, while a more recent study by Ganjisaffer *et al*.^15^ in the same area found resistance ratios between 10.7 and 16.9. Van Timmeren *et al*.^16^ also observed reductions in susceptibility to malathion and spinetoram over a 3‐year period of exposure in a Michigan population. These reports, along with continued reliance on chemical control and the fact that *D. suzukii* can complete 13 generations per year in many northern states, has prompted several laboratory selection trials to determine the likelihood of future resistance development. Smirle *et al*.^17^ found little evidence of resistance to malathion in two populations of *D. suzukii* collected in British Columbia, Canada after 20–30 generations of exposure but did observe variations in the 50% and 95% lethal concentration (LC_50_ and LC_95_) values between generations. Conversely, Gress and Zalom^14^ saw increases in spinosad LC_50_ values in a California field population selected for only five generations, resulting in resistance ratios of 1.15 for males and 1.11 for females. Most recently, Disi and Sial^18^ were able to generate spinosad‐ and malathion‐resistant populations through selection of a Georgia field population for 10–11 generations. Their heritability values also predicted that the development of ten‐fold resistance to malathion would occur within 37 generations but take only nine generations for spinosad. The seemingly high risk of spinosad resistance development across studies is particularly concerning given that it is one of only a few organic insecticides available for *D. suzukii* management.^18^

In this study, we investigated the propensity of three different Minnesota field populations with variable exposure histories to develop resistance to two commonly‐used conventional insecticides, the pyrethroid zeta‐cypermethrin, and the spinosad spinetoram. We selected for resistance through ten rounds of increasing exposure across 7–11 months. While laboratory selection studies on resistance development are increasing for *D. suzukii*, no data are currently available for zeta‐cypermethrin and spinetoram, two of the most frequently used insecticides in Minnesota.^7^ As such, these data will expand our knowledge about the threat of resistance to additional chemistries. This is also the first study to assess resistance in a Minnesota *D. suzukii* population that, when compared to field populations in other studies,^19^ will allow for broader comparisons between fly populations in different regions. Overall, this work will contribute useful information that can improve our ability to make predictions about resistance development in *D. suzukii* and inform current management strategies to maintain the integrity of current chemical controls and slow the development of resistance.

## 2 MATERIALS AND METHODS

### 2.1 Fly cultures

Three *D. suzukii* cultures were established by collecting infested raspberries at three different Minnesota sites along a north–south transect. The southernmost site was Wold Strawberries (hereafter referred to as the W population), a small berry operation near Mabel, MN. The intermediate site, the University of Minnesota's UMORE Station near Rosemount, MN (R population), was 147 miles north of Wold Strawberry. The northernmost site was a fruit‐growing operation called the Berry Patch near Forest Lake, MN (B population) and it was 46 miles north of UMORE Station. Each laboratory colony was established by collecting pupae from infested raspberry samples, which were collected on July 25, 2019 for the W population, September 14, 2019 for the R population, and August 1, 2019 for the B population. Eclosing adults were reared on a standard cornmeal‐based oligidic diet (cornmeal, sugar, agar, nutritional yeast, propionic acid, methyl paraben, ethanol) in narrow polystyrene vials (Genesee Scientific Corporation, San Diego, CA, USA).^20^ After each population was established, they were split into five different sub‐populations: one control that was not exposed to any insecticide, two sub‐populations that were exposed to zeta‐cypermethrin Mustang Maxx®, and two sub‐populations exposed to spinetoram Delegate®WG insecticides. Each sub‐population started with 20 vials containing approximately 50 flies per vial. Flies in each vial were transferred to fresh diet three times a week and empty vials were kept for new flies to emerge from 2 weeks later. This schedule produced 60 vials of new flies each week, and of these 60, 20 vials were kept and maintained each for each sub‐population. All colonies were maintained in a walk‐in growth chamber at ambient laboratory temperature, which ranged from 20 to 22 °C, under a 14 h:10 h light–dark cycle.

### 2.2 Insecticides

We chose two insecticides for the exposure treatments: Mustang Maxx® and Delegate®WG. Both insecticides are commonly used to control *D. suzukii* infestation in various berry cultivars.^21^ Mustang Maxx®, which is a broad‐spectrum pyrethroid (IRAC Group 3A) produced by FMC Corporation (Philadelphia, PA, USA), contains zeta‐cypermethrin as its active ingredient. Zeta‐cypermethrin is a synthetic compound that acts as a sodium channel modulator that kills insects by keeping sodium channels open and blocking nerve signals.^22^ The efficacy of Mustang Maxx® for *D. suzukii* control is quite high, as it maintains reasonably high residual contact against adults under field conditions. However, issues with ensuring effective contact with adult *D. suzukii* have been observed in field‐applications, particularly structurally‐complex cultivars such as caneberries.^23^, ^24^, ^25^ Spinetoram (Delegate® WG), produced by Corteva Agriscience (Indianapolis, IN, USA), is a fermentation product of the soil bacterium *Saccharopolyspora spinosa* and an analog to spinosad (IRAC Group 5) insecticides.^26^ It causes morbidity by disrupting nicotinic/gamma amino butyric acid (GABA)‐gated chloride channels.

### 2.3 Exposure protocol

Before the selection trials, dose–response assays were conducted for both insecticides for each field‐population. These were done on the field‐populations before they were split into their respective sub‐populations. Newly‐eclosed flies (24–48 h old) were anaesthetized with carbon dioxide (CO_2_) and placed in glass scintillation vials that were coated with a specific concentration of insecticide, prepared according to the methods in Van Timmeren *et al*.^27^ Six concentrations of Mustang Maxx® (FMC Corporation), 0, 0.05, 0.1, 0.5, 1, 20 ppm, and eight concentrations of Delegate®WG (Corteva Agriscience), 0, 0.001, 0.01, 0.1, 1, 10, 100, 1000 ppm, were tested. The concentrations refer to the ppm of the active ingredients. The Mustang Maxx® (0.8 lb AI/gal) formulation was mixed with acetone to obtain the earlier concentrations. The Delegate®WG (250 g AI/kg) formulation was mixed with water to achieve the earlier concentrations. Six replicate vials were used for each concentration and each vial contained three male and three female flies. Mortality was recorded after 4 h of exposure. Flies were recorded as dead if they were not moving or could not right themselves to standing within 10 s of falling. The results of these initial assays were used to create a dose–response curve and to calculate the exposure concentrations used in the selection treatments.

To select for resistance to each insecticide, flies from both sub‐populations were exposed to increasing concentrations of Mustang Maxx® and Delegate®WG. This was done by transferring all the newly‐eclosed flies from one diet vial into a scintillation vial coated with a specific concentration of insecticide, leaving them in the vial for 4 h, and then transferring them to a new vial with fresh diet. Twenty vials from each sub‐population were exposed at a time and always at the beginning of the week. The other 40 vials were saved as backups. After exposure vials were maintained by the schedule and protocol described earlier. Given that the sub‐populations were established at different times, the dates and concentrations for each were different. Supporting Information, Table S1 shows the dates of exposure, the number of exposures, and the range of concentrations used for each insecticide and sub‐population. Initial exposures started in the LC_5_–LC_10_ dose range but were modulated up and down based on survivorship after exposure. Exposures were attempted every week, however, often times mortality from exposure would be higher than expected and vial populations would be given time to recuperate. In some cases, survivorship of the exposed flies was too low to maintain the population, in which case, 20 backup vials that were not initially selected for exposure were used to maintain the colony until the next exposure. Flies from different vials were never mixed, thus each vial represented a unique mating population.

### 2.4 Data analysis

A probit analysis was performed using the data from the initial dose–response bioassays to determine the sub‐lethal concentrations for each insecticide. Nonlinear curve‐fitting was done in GraphPad Prism 9.1.2 for Windows (GraphPad Software, San Diego, CA, USA) to create the initial and final dose–response curves for each population. An extra sum‐of‐squares *F* test, also performed in GraphPad Prism 9.1.2, was used to compare two dose–response curve parameters (LC_50_ and Hill slope) between the different field populations (initial dose–response curves), including *post hoc* comparison where needed (using an adjusted Bonferroni‐corrected *P*‐value). This test was also used to compare control and exposed sub‐population curve parameters for each insecticide. Differences in the LC_50_, the top concentration (*y* value where the upper part of the curve plateaus), and the Hill slope of each curve were analyzed to detect statistical differences between the resultant dose–response curves for the control and exposed sub‐populations after approximately ten rounds of exposure. Resistance ratios were also calculated, by dividing the LC_50_ value of each sub‐population by that of their control, to quantify the magnitude of change in LC_50_ value after exposure.

## 3 RESULTS

### 3.1 Initial dose–response assays

**Figure 1:**
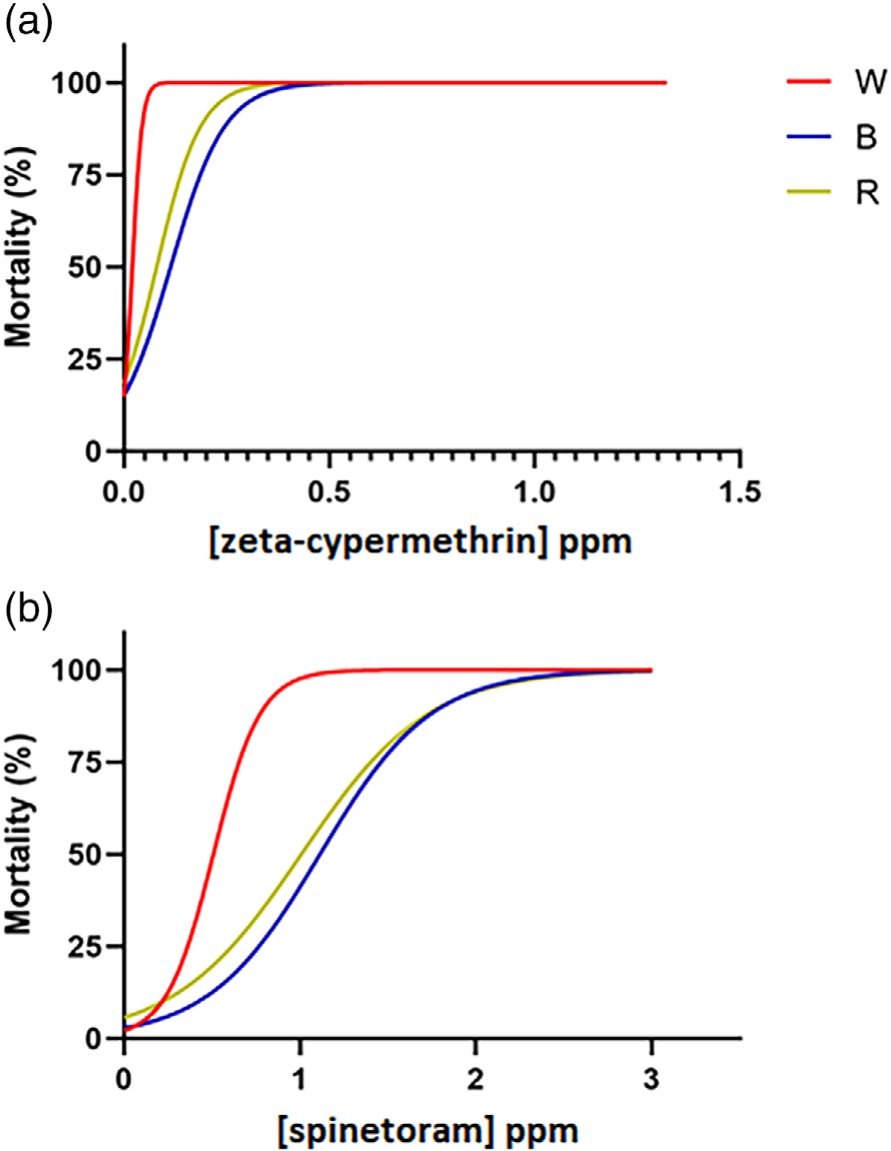
Initial dose–response curves for each field population (W, B, and R) exposed to (a) zeta‐cypermethrin and (b) spinetoram insecticides.

Significant differences in the initial dose–response curves were apparent across the three field populations for both insecticides. Table 1 shows that the LC_50_ values varied across populations for zeta‐cypermethrin and spinetoram, while the Hill slope only varied significantly for spinetoram. There were no differences in top mortality limits across populations for either insecticide (Table 1). The initial susceptibilities of the R and B populations were statistically similar across both insecticides, while the W population was significantly different from both (Tables 1 and S2). Figure 1 shows that the W population, which was the southernmost site sampled, was much more susceptible to both insecticides than either the R and B populations, exhibiting significantly lower LC_50_ values and higher Hill slopes (Table S2).

**Table 1:** Extra sum‐of‐squares *F* test (no Hill Slope constraint) results showing statistical differences for the initial dose–response characteristics across field populations and the *post hoc* contrasts

	LC_50_			Top mortality			Hill slope		
Insecticide	df	*F*	*P*‐Value	df	*F*	*P*‐Value	df	*F*	*P*‐Value
Zeta‐cypermethrin	2, 48	22.74	**<0.0001**	2, 42	0.051	0.9508	2, 48	0.039	0.9612
W *versus* R	1, 32	17.11	**0.0002**	1, 28	0.076	0.7843	1, 32	0.074	0.7877
W *versus* B	1, 32	45.93	**<0.0001**	1, 28	0.001	0.9899	1, 32	0.056	0.8145
R *versus* B	1, 32	2.98	0.0941	1, 28	0.057	0.8133	1, 32	0.012	0.9138
Spinetoram	2, 57	7.08	**0.0018**	2, 51	0.838	0.4383	2, 57	7.618	**0.0012**
W *versus* R	1, 38	11.21	**0.0018**	1, 34	1.266	0.2684	1, 38	11.99	**0.0013**
W *versus* B	1, 38	16.56	**0.0002**	1, 34	1.140	0.2932	1, 38	5.119	0.0295
R *versus* B	1, 38	1.95	0.1712	1, 34	0.039	0.8443	1, 38	0.977	0.3291

Note: bolded values indicate significance at *P* ≤ 0.05 (a Bonferroni‐adjusted *P* value of 0.0125 was used for to all *post hoc* contrasts). LC_50_, median lethal concentration.

### 3.2 Selection trials

#### 3.2.1 Zeta‐cypermethrin

**Figure 2:**
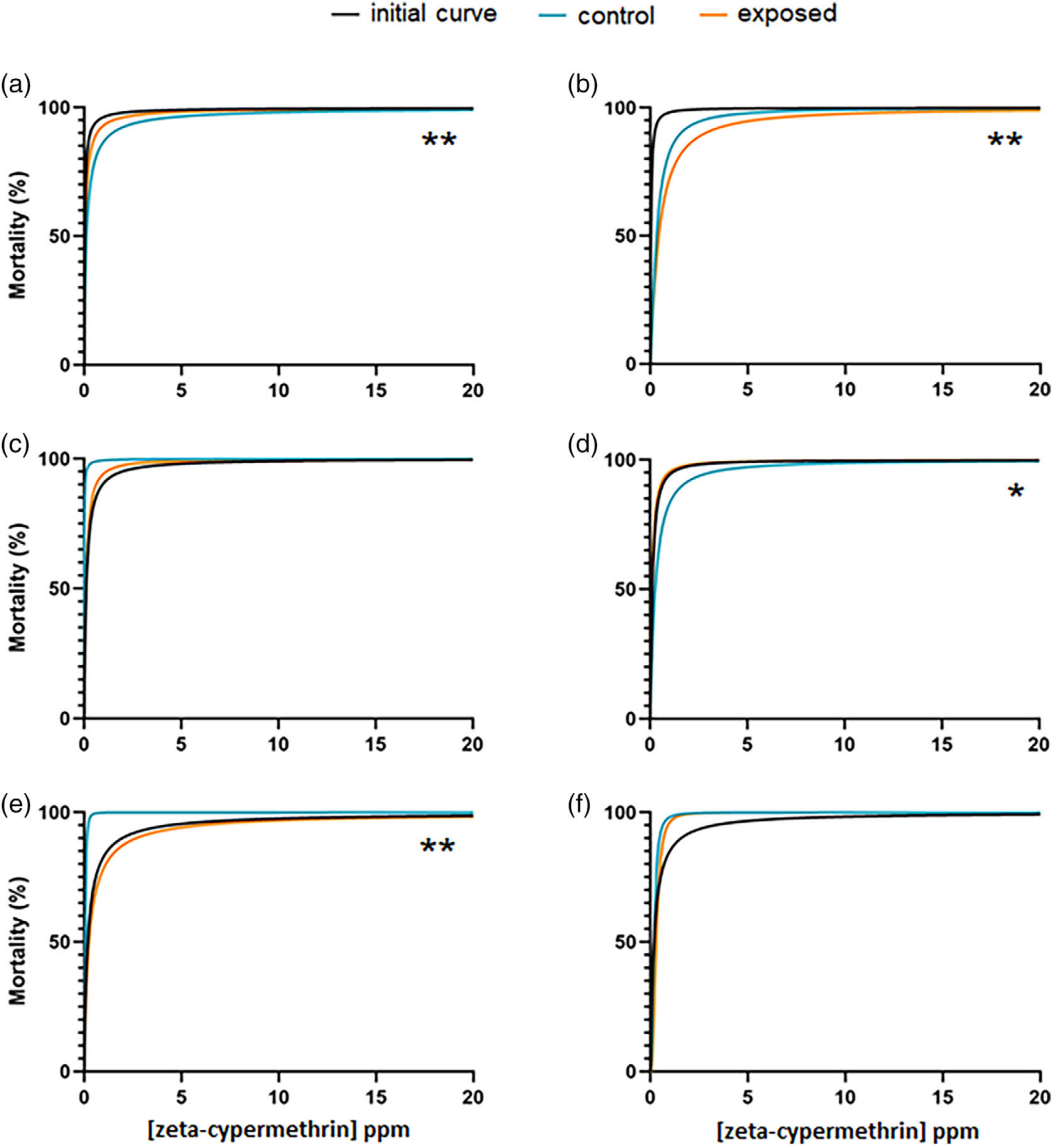
Dose–response curves for the initial bioassays, the control, and the exposed sub‐populations for the W (a, b), R (c, d), and B (e, f) field‐populations exposed to zeta‐cypermethrin. The first column shows the curves for sub‐populations 1 and the second column for sub‐populations 2. Asterisks indicate significant differences in LC_50_ values between the control and exposed sub‐population at *P* ≤ 0.05 (see Table 2).

After ten rounds of increasing exposure to zeta‐cypermethrin, the LC_50_ values of three control populations and two exposed populations diverged in from their initial dose–responses. Table 2 shows that the W population controls and both exposed sub‐populations had significantly lower LC_50_ values than the initial field population assayed (Table S3; Fig. 2(a,b)). The dose–response curve for the W sub‐population 2 also had a significantly higher Hill slope than the initial dose–response curve (Tables 2 and S3). Both parameters suggest that by the end of the exposure period these populations were more susceptible than the population initially bioassayed. However, no statistical difference was found between the LC_50_ of the controls and either exposed sub‐population (Tables 2 and S3).

**Table 2:** Extra sum‐of‐squares statistics showing whether the curve parameters [median lethal concentration (LC_50_) values, top mortality values, and the Hill slopes] were significantly different in comparisons between the initial dose–response bioassays and the control (C) and sub‐population (W, R, and B) after the zeta‐cypermethrin exposure period

Contrast	Population		LC_50_			Top mortality			Hill slope		
			df	*F*	*P*‐Value	df	*F*	*P*‐Value	df	*F*	*P*‐Value
Initial *versus* control	W	C	1, 28	5.118	**0.0316**	1, 28	0.001	0.9729	1, 28	0.225	0.6392
		C	1, 28	6.787	**0.0145**	1, 28	0.134	0.7176	1, 28	1.903	0.1786
	R	C	1, 28	1.016	0.3222	1, 28	0.279	0.6018	1, 28	4.113	0.0522
		C	1, 28	1.730	0.1990	1, 28	0.512	0.4804	1, 28	0.996	0.3269
	B	C	1, 28	13.74	**0.0009**	1, 28	0.080	0.7806	1, 28	4.515	**0.0426**
		C	1, 28	0.401	0.5319	1, 28	0.071	0.7924	1, 28	9.075	**0.0054**
Initial *versus* exposed	W	1	1, 28	13.04	**0.0012**	1, 28	0.002	0.9627	1, 28	0.391	0.5369
		2	1, 28	4.784	**0.0372**	1, 28	0.053	0.8192	1, 28	6.419	**0.0172**
	R	1	1, 28	0.165	0.6872	1, 28	0.138	0.7134	1, 28	1.592	0.2175
		2	1, 28	0.739	0.3971	1, 28	0.768	0.3882	1, 28	6.621	**0.0157**
	B	1	1, 28	3.713	0.0642	1, 28	0.030	0.8642	1, 28	5.089	**0.0321**
		2	1, 28	0.881	0.3540	1, 28	1.416	0.2441	1, 28	10.09	**0.0036**
Control *versus* exposed	W	1	1, 28	0.977	0.3313	1, 28	0.005	0.9457	1, 28	0.009	0.9267
		2	1, 28	3.481	0.0726	1, 28	0.004	0.9499	1, 28	2.445	0.1291
	R	1	1, 28	1.091	0.3052	1, 28	0.063	0.8030	1, 28	1.695	0.2036
		2	1, 28	6.230	**0.0187**	1, 28	0.015	0.9039	1, 28	3.970	0.0562
	B	1	1, 32	6.902	**0.0131**	1, 28	0.001	0.9861	1, 28	3.186	0.0851
		2	1, 28	0.041	0.8420	1, 28	0.897	0.3517	1, 28	0.071	0.792

Note: bolded values indicate significance at *P* ≤ 0.05.

The dose–response curves for the control and exposed R sub‐population 1 were statistically similar to the initial bioassay curve, but there were some significant differences between the control and exposed R sub‐populations. Exposed R sub‐population 2 showed a significantly higher Hill slope than the initial curve and a lower LC_50_ value than the control curve (Tables 2 and S3). This shows that exposed R sub‐population 2 became more susceptible than the control after ten rounds of exposure (Table S3; Fig. 2(c,d)). There were no differences in curve parameters between R sub‐population 1, the control, or initial curves (Table S3; Fig. 2(c,d)).

There were some differences between the initial dose–response curve and the controls for the B population. One of the B control populations showed a significantly lower LC_50_ and a higher Hill slope than the initial dose–response curve, while the other control population only had a significantly higher Hill slope but a similar LC_50_ (Tables 2 and S3). The LC_50_ values of the exposed B sub‐populations were not different from the initial curve (Table 2), but both Hill slopes were significantly larger (Table 2). Only exposed B sub‐population 1 exhibited a statistical difference between its LC_50_ value compared to the control population (Table 2), with the LC_50_ being higher for the exposed population (Table S3; Fig. 2(e,f)). This shows that B sub‐population 1 became less susceptible to zeta‐cypermethrin over the ten rounds of exposure compared to the control.

Table 3 shows the resistance ratios for each sub‐population. Three sub‐populations showed resistance ratios greater than 1, while the other three sub‐populations were less than 1. Only two sub‐population had significantly different LC_50_ values from the controls, thus statistically significant resistance ratios. The resistance ratio for B sub‐population 1 was 9.31, indicating that this population was almost ten times less susceptible to zeta‐cypermethrin than the control. The resistance ratio for R sub‐population 2 was 0.410, which was the lowest of any zeta‐cypermethrin exposure group, and shows that this sub‐population was more than two times more susceptible to zeta‐cypermethrin than the control.

**Table 3:** The resistance ratios (exposed/control values), based on the median lethal concentration (LC_50_) values

			Resistance ratio
Insecticide	Population		LC_50_
Zeta‐cypermethrin	W	1	0.545
		2	1.85
	R	1	1.23
		2	**0.410**
	B	1	**9.31**
		2	0.741
Spinetoram	W	1	0.110
		2	**1.55**
	R	1	0.386
		2	0.362
	B	1	0.794
		2	**0.687**

Note: bolded values show the ratios for the sub‐populations that had significantly different LC_50_ values from control (*P* ≤ 0.05).

#### 3.2.2 Spinetoram

**Figure 3:**
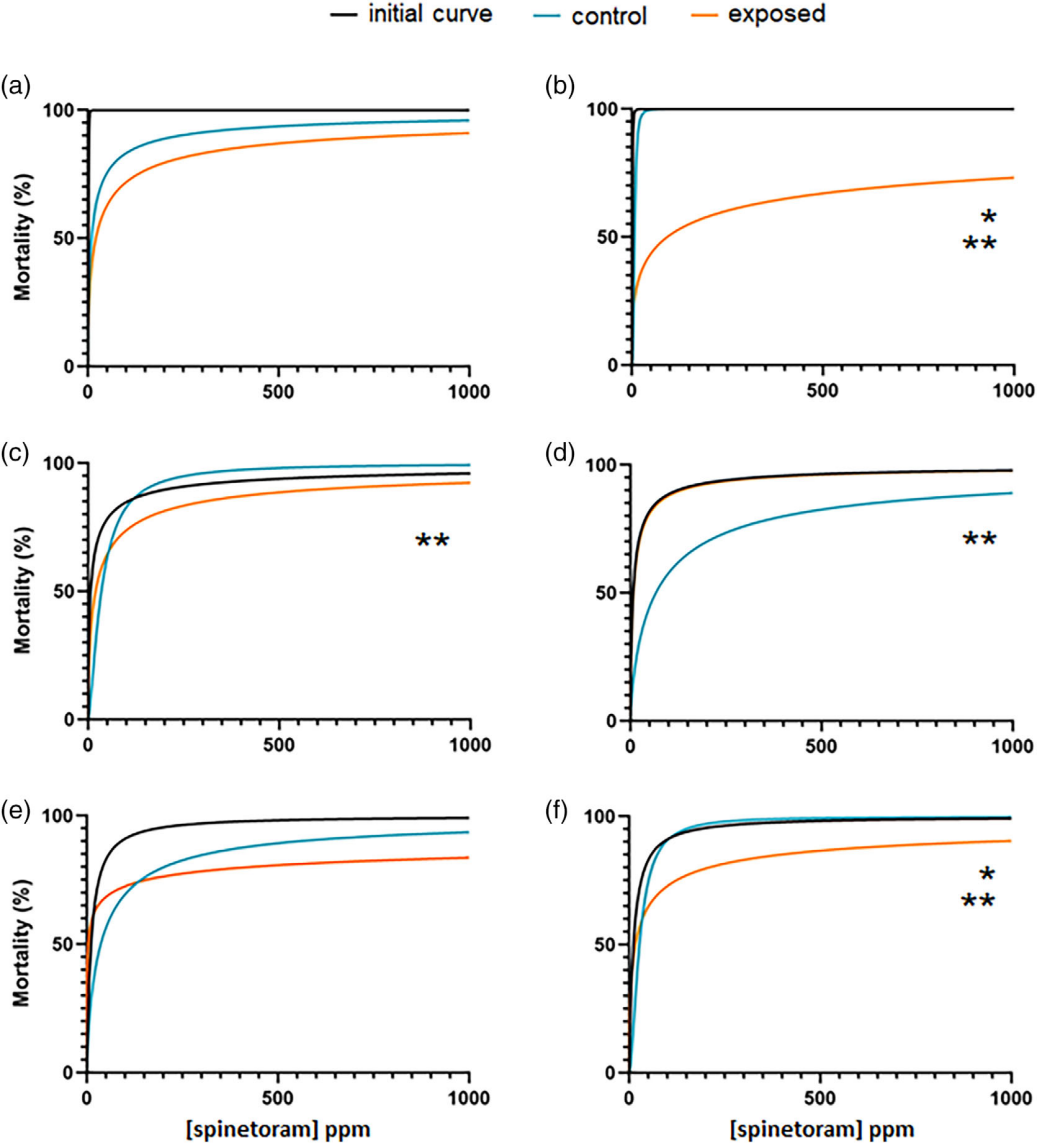
Dose–response curves for the initial bioassays, the control, and the exposed sub‐populations for the W (a, b), R (c, d), and B (e, f) field‐populations exposed to spinetoram. The first column shows the curves for sub‐populations 1 and the second column for sub‐populations 2. Asterisks indicate significant differences in LC_50_ values (*) and/or top mortality values (**) between the control and exposed sub‐population at *P* ≤ 0.05 (see Table 3).

The W population control and exposed sub‐population LC_50_ values did diverge significantly from the initial curve (Table 4). The control and both exposed sub‐populations had higher LC_50_ values than the initial curve (Table S4; Fig. 3(a,b)). However, only W sub‐population 2 showed significant differences from the control after approximately ten rounds of exposure to spinetoram (Table 4), with the LC_50_ being higher and top concentration being lower than the control (Table S4; Fig. 3(b)).

**Table 4:** Extra sum‐of‐squares statistics showing whether the curve parameters [median lethal concentration (LC_50_) values, top mortality values, and the Hill slopes] were significantly different in comparisons between the initial dose–response bioassays and the control (C) and sub‐population (W, R, and B) after the spinetoram exposure period

Contrast	Population		LC_50_			Top mortality			Hill slope		
			df	*F*	*P*‐Value	df	*F*	*P*‐Value	df	*F*	*P*‐Value
Initial *versus* control	W	C	1, 34	11.72	**0.0016**	1, 34	1.046	0.3136	1, 34	0.233	0.6324
		C	1, 34	12.56	**0.0012**	1, 34	1.237	0.2738	1, 34	0.026	0.8730
	R	C	1, 34	2.355	0.1341	1, 34	3.821	0.0589	1, 34	4.669	**0.0378**
		C	1, 34	4.960	**0.0327**	1, 34	4.448	**0.0424**	1, 34	0.816	0.3727
	B	C	1, 34	4.146	**0.0496**	1, 34	0.001	0.9784	1, 34	1.770	0.1922
		C	1, 34	<0.001	0.9942	1, 34	6.27	**0.0172**	1, 34	2.645	0.1131
Initial *versus* exposed	W	1	1, 34	12.97	**0.0010**	1, 34	0.375	0.5441	1, 34	2.780	0.1047
		2	1, 34	16.71	**0.0003**	1, 34	36.42	**<0.0001**	1, 34	<0.001	0.9944
	R	1	1, 38	2.644	0.1122	1, 34	11.71	**0.0016**	1, 38	3.638	0.0641
		2	1, 34	0.151	0.7000	1, 34	0.014	0.9063	1, 34	0.006	0.9373
	B	1	1, 34	0.764	0.3882	1, 34	2.456	0.1263	1, 34	1.446	0.2375
		2	1, 34	1.729	0.1973	1, 34	26.91	**<0.0001**	1, 34	2.078	0.1586
Control *versus* exposed	W	1	1, 34	2.060	0.1604	1, 34	0.001	0.9803	1, 34	0.325	0.5725
		2	1, 34	27.78	**<0.0001**	1, 34	41.38	**<0.0001**	1, 34	0.016	0.8991
	R	1	1, 34	1.150	0.2914	1, 34	4.280	**0.0462**	1, 34	1.149	0.2913
		2	1, 34	3.974	0.0543	1, 34	4.743	**0.0364**	1, 34	0.743	0.3947
	B	1	1, 34	0.067	0.7974	1, 34	0.674	0.4173	1, 34	6.329	**0.0168**
		2	1, 34	6.553	**0.0151**	1, 34	7.68	**0.0090**	1, 34	<0.01	0.9910

Note: bolded values indicate significance at *P* ≤ 0.05.

For the R population, there were significant differences in the LC_50_ values and top concentrations between the controls and the initial dose–response curve but not the exposed sub‐populations (Table 4). One of the controls exhibited a lower LC_50_ the initial curve value, while the other control had a lower top concentration than the initial curve (Table S4). The exposed R sub‐population 1 also showed a significantly lower top concentration than the initial curve (Table S4; Fig. 3(c)) but a similar LC_50_ (Table 4). Only the exposed R sub‐population 2 showed significant differences in LC_50_ and top concentration compared to the controls (Table 4; Fig. 3(d)), with the exposed sub‐population having a significantly lower LC_50_ and a higher top concentration than the controls (Table S4). The top concentration of the exposed R sub‐population 1 was different from the control (Table 4), with the exposed sub‐population plateauing at a significantly lower concentration (Table S4; Fig. 3(c)).

For the B population, one of the controls exhibited an LC_50_ that varied from the initial curve, being significantly larger (Table S4), and the other control curve had a top concentration that was (Table 4) significantly lower than the initial curve (Tables 4 and S4). The LC_50_ values of the exposed sub‐populations were statistically similar to the initial curve (Table 4), while the top concentration of B sub‐population 2 was significantly lower (Table S4; Fig. 3(f)). The LC_50_ and top concentration of B sub‐population 2 were both significantly lower than the control curve, while there were no differences between the control and sub‐population 1 (Tables 4 and S4). Despite this, the Hill slope of the exposed sub‐population 1 curve was significantly higher than the control (Table 4).

For the spinetoram exposures, only one sub‐population, W sub‐population 2, had a resistance ratio greater than 1, while all other ratios were less than 1 (Table 3). Of the six sub‐populations exposed, only three showed significant differences from the controls, with the W sub‐population 2 becoming 1.5 times less susceptible than the controls, the R sub‐population 2 becoming almost three times more susceptible than controls, and the B sub‐population 2 approximately 1.5 times more susceptible.

## 4 DISCUSSION

When identifying indicators of resistance development, one can explore several different aspects of susceptibility. In this study, we focused on changes in dose–response characteristics of the LC_50_ value, top mortality limit, and the Hill slope of the curves for sub‐populations that were exposed to increasing concentrations of insecticide. The LC_50_ specifies the dose of insecticide needed to kill 50% of the population, while the top mortality limit indicates point at which mortality plateaus. Increases in the LC_50_ of exposed *versus* control populations indicates a higher dose requirement for the same level of mortality and can indicate the development of insecticide resistance. Reductions in the top mortality limit for exposed *versus* control populations may also indicate resistance development, as this value can show whether maximum mortality is being achieved. The Hill slope shows the steepness of the curve and the intensity of response to the insecticide. Decreases in the Hill slope may provide evidence for resistance development but only when the top mortality limits of the exposed and control populations are similar, as it is possible for curves with different maximum mortality to have the same Hill slope. Because these parameters describe different aspects of susceptibility, they were assessed separately and in combination to determine the likelihood of resistance development in our sub‐populations.There were five potential instances of resistance evolution based on increases in the LC_50_ and/or the top mortality limit of the exposed sub‐populations compared to the controls. The first potential case of resistance was in B sub‐population 1, which exhibited an almost ten‐fold increase in LC_50_ value for zeta‐cypermethrin relative to the control population (Table 3). However, the curve for the exposed population did not differ from the initial population curve, indicating that the increase in LC_50_ was largely due to the control population becoming more susceptible over the exposure period, and thus, making support for resistance development questionable. The second was W sub‐population 2, which was 1.5 times less susceptible to spinetoram than the controls after the exposure period (Table 3). In addition to the impacts on LC_50_ value, three sub‐populations showed significant decreases in the top mortality limit of exposed sub‐populations compared to controls, indicating that they had lower overall lethality at the highest concentration tested. W sub‐population 2 showed a 33.0% reduction in the top mortality limit compared to controls, and R sub‐population 1 exhibited an 18.0% decrease in the top mortality limit for spinetoram (Table S4). B sub‐population 2, despite showing a significantly lower LC_50_ than controls, also had a 16.7% decrease in the top mortality limit for spinetoram (Table S4).

There was little evidence for resistance in the zeta‐cypermethrin treatments, as the Hill slopes and the top mortality limits did not vary substantially between the controls and the exposed sub‐populations. These parameters were more variable for the spinetoram treatments. All three parameters differed, but the top morality limits were the most variable. Four of the six spinetoram sub‐populations had significantly different top mortality limits compared to the controls, with the majority having lower limits, suggesting that selection had a stronger overall impact on the spinetoram than the zeta‐cypermethrin treatments.

In seven out of 12 instances, the dose–response of the control sub‐populations diverged significantly from the initial dose–response curves after the exposure period. Although unexpected, this is perhaps not surprising, considering that the control dose–response bioassays were performed between 1 and 1.66 years after the initial bioassays (Table 1), allowing changes in susceptibility to develop over time. It is interesting, however, that the patterns were very consistent within each insecticide but very different across insecticides. For instance, of the three zeta‐cypermethrin controls that varied, all developed lower LC_50_ values and two showed higher Hill slopes than the initial curves (Table S2), suggesting that the control populations became more susceptible over time. Conversely, of the four spinetoram controls that had significantly different LC_50_ values, all were higher than the initial values, and two sub‐population controls had lower top mortality limits, suggesting that the spinetoram control populations became less susceptible. Curiously, these changes occurred despite no exposure to insecticide. The most likely explanation for this is that adaptation of the field populations to their new laboratory setting had some unexpected and unintended impacts on genes related to insecticide tolerance. These could be genes associated with detoxification, cellular repair, cuticular integrity, or general movement patterns. It is also possible that the laboratory environment, particularly the switch to artificial diet with antimicrobial components, could have impacted the microbiome of the flies in ways that indirectly affected susceptibility.^28^, ^29^, ^30^, ^31^ For instance, microbial associations have been found to impact insecticide detoxification and resistance in other insect species.^32^, ^33^, ^34^ Differences in microbiome communities have even been documented in laboratory fly colonies that were fed the same diets for over 50 generations, suggesting that other non‐nutritional factors can also affect microbial community structure within laboratory cultures.^35^ Unfortunately, the factors that contributed to the observed differences in our control populations remain unknown.

We observed a similar pattern of differences in the initial curve and exposed sub‐populations at the end of the exposure period. In two instances, both W sub‐populations, the LC_50_ was significantly lower for the zeta‐cypermethrin exposed treatment compared to the initial curve, while the Hill slope was higher in four cases (Table S2). This suggests an increase in susceptibility. For spinetoram, two sub‐populations, again both W sub‐populations, had higher LC_50_ values and three had lower top mortality limits (Table S3), suggesting lower susceptibility. In these cases, it is difficult to determine whether these differences are due to laboratory adaptation or insecticide exposure, as both may have occurred. Although both control and exposed sub‐populations were subject to the same laboratory environment, it is possible that these pressures impacted the populations differently. In cases where the control populations were impacted but the exposed sub‐population were not, it can affect our ability to accurately identify resistance. For instance, B sub‐population 1 had a zeta‐cypermethrin LC_50_ value that was similar to the initial curve, while its control population had a LC_50_ value that was lower than the initial curve. When comparing the LC_50_ values of the control and exposed sub‐population, the reduced susceptibility of the control could make the exposed sub‐population appear more resistant, while it may simply be the case that laboratory selection has reduced the susceptibility of the control but not the comparator. For the W sub‐population 2, which was the only sub‐population to show an increase in LC_50_ for spinetoram, both the control and exposed sub‐population had significantly higher LC_50_ values than the initial curve, making the differences between the control and exposed populations less likely to be confounded by potential laboratory selection.

After accounting for how these changes in control parameters may have impacted the accuracy of our results, the evidence for resistance to zeta‐cypermethrin in B sub‐population 1 must be further evaluated. The evidence for spinetoram resistance is, however, more robust, as all instances of resistance were either not associated with strong differences between the initial and control dose–response parameters, or were associated with similar differences in the initial curve and both control and exposed group curves (Table S3). In light of this, our results show little evidence for resistance development to zeta‐cypermethrin across field populations, despite some differences in susceptibility. Resistance to spinetoram, however, is evident, as three of the six spinetoram sub‐populations had higher LC_50_ values than the controls (only two were significantly higher) and five of the six sub‐populations had lower top mortality limits than the controls (only three were significantly lower).

Despite only detecting evidence for resistance in three sub‐populations (i.e. LC_50_ increase in zeta‐cypermethrin B sub‐population 1 and spinetoram W sub‐population 2, and reduced top mortality limit in spinetoram R sub‐population 1), the fact that the exposed sub‐populations persisted across an 8‐ to 45‐fold increase in exposure over just ten rounds of selection suggests that field populations of *D. suzukii* have a considerable propensity to develop resistance to selected insecticides under laboratory selection conditions. A few other instances of resistance to spinosyns, such as spinosad and spinetoram, have been reported in *D. suzukii*,^16^, ^17^ and our results are consistent with Disi and Sial's^18^ data, showing resistance development in a field‐caught *D. suzukii* population to spinosad in 10–11 generations of laboratory selection.

In conclusion, given the continued reliance on chemical controls for *D. suzukii* management, the risk of resistance development in local populations is likely to continue. While progress is being made in the optimization of chemical controls, as well as the development of biorational insecticides or biopesticides^36^, ^37^, ^38^, ^39^ and the non‐chemical options,^6^, ^40^, ^41^, ^42^, ^43^, ^44^, ^45^ insecticide rotation and proper integrated pest management (IPM) practices will be key to slowing the spread of resistance. While our data suggest that field populations of *D. suzukii* are capable of developing resistance to some insecticides in rather short periods of time, much more work is needed to better understand the genetic factors related to insecticide resistance in *D. suzukii*, as well as the distribution of resistance alleles in field populations. Laboratory selection studies and resistance monitoring will be key in this pursuit.^19^

## CONFLICT OF INTEREST

All authors have read and approved this version of the article, and declare no conflict of interest.

## Supporting information


**Table S1.** Summary of the selection process for each initial population, sub‐population, and insecticide, including start date (date of collection for the initial population), number of exposures, dose range (ppm), and final bioassay date. For the final bioassays, all control populations were assayed at the same time as their corresponding sub‐population and exposed to the same dose ranges.
**Table S2.** The median lethal concentration (LC_50_) values, top mortality values, and Hill slopes for the initial dose–response curves for each field population and insecticide. Different letters indicate significant differences between populations at *P* ≤ 0.05.
**Table S3.** The dose–response characteristics for the initial bioassay, as well as the controls and exposure sub‐population curves after the zeta‐cypermethrin exposure period. Different letters indicate significant differences between initial, control, and exposed sub‐population values at *P* ≤ 0.05.
**Table S4.** The dose–response characteristics for the initial bioassay, as well as the controls and exposure sub‐population curves after the spinetoram exposure period. Dashes represent undefined parameters. Different letters indicate significant differences between initial, control, and exposed sub‐population values at *P* ≤ 0.05.

## Data Availability

The data that support the findings of this study are openly available in Data Repository of U of M at https://hdl.handle.net/11299/226613.
