# Supplementary material for: Propensity for resistance development in the invasive berry pest, spotted‐wing drosophila (Drosophila suzukii), under laboratory selection
Source: Pest Manag Sci. 2022 Aug 31;78(12):5203–12. doi: 10.1002/ps.7139 (PMC9804391; doi:10.1002/ps.7139)
Supplement: Supplementary file 1 — Table S1. Summary of the selection process for each initial population, sub‐population, and insecticide, including start date (date of collection for the initial population), number of exposures, dose range (ppm), and final bioassay date. For the final bioassays, all control populations were assayed at the same time as their corresponding sub‐population and exposed to the same dose ranges. Table S2. The median lethal concentration (LC50) values, top mortality values, and Hill slopes for the initial dose–response curves for each field population and insecticide. Different letters indicate significant differences between populations at P ≤ 0.05. Table S3. The dose–response characteristics for the initial bioassay, as well as the controls and exposure sub‐population curves after the zeta‐cypermethrin exposure period. Different letters indicate significant differences between initial, control, and exposed sub‐population values at P ≤ 0.05. Table S4. The dose–response characteristics for the initial bioassay, as well as the controls and exposure sub‐population curves after the spinetoram exposure period. Dashes represent undefined parameters. Different letters indicate significant differences between initial, control, and exposed sub‐population values at P ≤ 0.05. [file PS-78-5203-s001.docx]

SI Table 1. Summary of the selection process for each initial population, sub-population, and insecticide, including start date (date of collection for the initial population), number of exposures, dose range (ppm), and final bioassay date. For the final bioassays, all control populations were assayed at the same time as their corresponding sub-population and exposed to the same dose ranges.

| Population |  | zeta-cypermethrin | | | | spinetoram | | | |
| --- | --- | --- | --- | --- | --- | --- | --- | --- | --- |
|  | Start Date | # Exposures | Start Dose | End Dose | Date Assayed | # Exposures | Start Dose | End Dose | Date Assayed |
| W |  |  |  |  |  |  |  |  |  |
| initial | 7-25-19 | 1 | 0 | 20 | 8-26-19 | 1 | 0 | 1000 | 8-23-19 |
| sub-pop 1 | 6-5-20 | 10 | 0.005 | 0.40 | 2-12-21 | 8 | 0.1 | 4.5 | 4-22-21 |
| sub-pop 2 | 7-7-20 | 10 | 0.005 | 0.55 | 3-1-21 | 10 | 0.8 | 6.5 | 2-26-21 |
| R |  |  |  |  |  |  |  |  |  |
| initial | 9-14-19 | 1 | 0 | 20 | 1-11-20 | 1 | 0 | 1000 | 1-11-20 |
| sub-pop 1 | 6-15-20 | 10 | 0.02 | 0.40 | 4-22-21 | 9 | 0.5 | 16.5 | 4-15-21 |
| sub-pop 2 | 7-7-20 | 10 | 0.04 | 0.45 | 2-1-21 | 10 | 2 | 17.9 | 4-15-21 |
| B |  |  |  |  |  |  |  |  |  |
| initial | 8-1-19 | 1 | 0 | 20 | 1-11-20 | 1 | 0 | 1000 | 1-11-20 |
| sub-pop 1 | 6-15-20 | 10 | 0.04 | 0.45 | 1-25-21 | 9 | 1 | 17.0 | 5-5-21 |
| sub-pop 2 | 7-7-20 | 10 | 0.02 | 0.55 | 2-1-21 | 9 | 1 | 17.2 | 4-14-21 |

SI Table 2. The LC_50_ values, top mortality values, and Hill slopes for the initial dose-response curves for each field population and insecticide. Different letters indicate significant differences between populations at P≤0.05.

| Insecticide | Pop | LC_50_ | | Top Mortality | | Hill Slope | |
| --- | --- | --- | --- | --- | --- | --- | --- |
| zeta-cypermethrin | W | 1.049 | (1.032-0.069) a | 102.4 | (92.7-120.6) a | 0.847 | (0.311-1.86) a |
|  | R | 1.201 | (1.097-1.310) b | 105.0 | (89.5-125.9) a | 0.987 | (0.505-1.96) a |
|  | B | 1.299 | (1.208-1.408) b | 102.5 | (88.9-119.3) a | 1.00 | (0.607-1.7) a |
| spinetoram | W | 3.221 | (und-4.109) a | 100.0 | (95.5-105.6) a | 5.96 | (1.71-und) a |
|  | R | 10.18 | (7.246-14.66) b | 107.3 | (94.7-130.0) a | 0.789 | (0.441-und) b |
|  | B | 12.99 | (10.16-16.90) b | 105.6 | (96.0-118.1) a | 1.01 | (0.630-und) ab |

SI Table 3. The dose-response characteristics for the initial bioassay, as well as the controls and exposure sub-population curves after the zeta-cypermethrin exposure period. Different letter indicate significant differences between initial, control, and exposed sub-population values at P≤0.05.

| Insecticide | Pop | | LC_50_ | | | | Top Mortality | | | | Hill Slope | | | |
| --- | --- | --- | --- | --- | --- | --- | --- | --- | --- | --- | --- | --- | --- | --- |
|  |  |  | control | | exp | | control | | exp | | control | | exp | |
| zeta-cypermethrin | initial | | 1.05 | a |  |  | 102.4 | a |  |  | 0.847 | a |  |  |
|  | W | 1 | 0.266 | b | 0.145 | b | 102.7 | a | 102.0 | a | 1.10 | ab | 1.15 | ab |
|  |  | 2 | 0.335 | b | 0.619 | b | 99.26 | a | 99.99 | a | 1.62 | ab | 4.01 | b |
|  | initial | | 0.137 | ab |  |  | 105.0 | a |  |  | 0.987 | a |  |  |
|  | R | 1 | 0.096 | ab | 0.118 | ab | 100.6 | a | 102.0 | a | 2.43 | ab | 1.51 | ab |
|  |  | 2 | 0.249 | a | 0.102 | b | 97.21 | a | 98.15 | a | 1.45 | ab | 2.01 | b |
|  | initial | | 1.30 | a |  |  | 102.5 | a |  |  | 1.00 | a |  |  |
|  | B | 1 | 0.060 | b | 0.559 | a | 100.2 | a | 100.0 | a | 2.46 | b | 25.09 | b |
|  |  | 2 | 0.409 | a | 0.303 | a | 100.0 | a | 92.33 | a | 12.9 | b | 4.22 | b |

SI Table 4. The dose-response characteristics for the initial bioassay, as well as the controls and exposure sub-population curves after the spinetoram exposure period. Dashes represent undefined parameters. Different letter indicate significant differences between initial, control, and exposed sub-population values at P≤0.05.

| Insecticide | Pop | | LC_50_ | | | | Top Mortality | | | | Hill Slope | | | |
| --- | --- | --- | --- | --- | --- | --- | --- | --- | --- | --- | --- | --- | --- | --- |
|  |  |  | control | | exp | | control | | exp | | control | | exp | |
| spinetoram | initial | | 3.22 | a |  |  | 100.0 | a |  |  | 5.96 | a |  |  |
|  | W | 1 | 13.38 | bc | 30.01 | bc | 94.75 | ab | 94.48 | ab | 1.64 | a | 11.1 | a |
|  |  | 2 | 6.90 | b | 10.70 | c | 96.73 | a | 65.00 | b | 2.59 | a | - | a |
|  | initial | | 10.18 | a |  |  | 107.3 | a |  |  | 0.789 | a |  |  |
|  | R | 1 | 28.85 | ab | 11.14 | b | 93.56 | a | 76.67 | b | 1.69 | b | - | ab |
|  |  | 2 | 33.96 | b | 12.31 | ab | 81.48 | b | 106.2 | a | 1.16 | ab | 0.814 | ab |
|  | initial | | 12.99 | ac |  |  | 105.6 | a |  |  | 1.01 | ab |  |  |
|  | B | 1 | 43.62 | b | 34.65 | ab | 106.1 | a | 93.38 | a | 0.619 | a | 1.93 | b |
|  |  | 2 | 12.29 | a | 8.45 | c | 90.0 | b | 75.00 | c | 9.50 | a | 4.97 | a |
